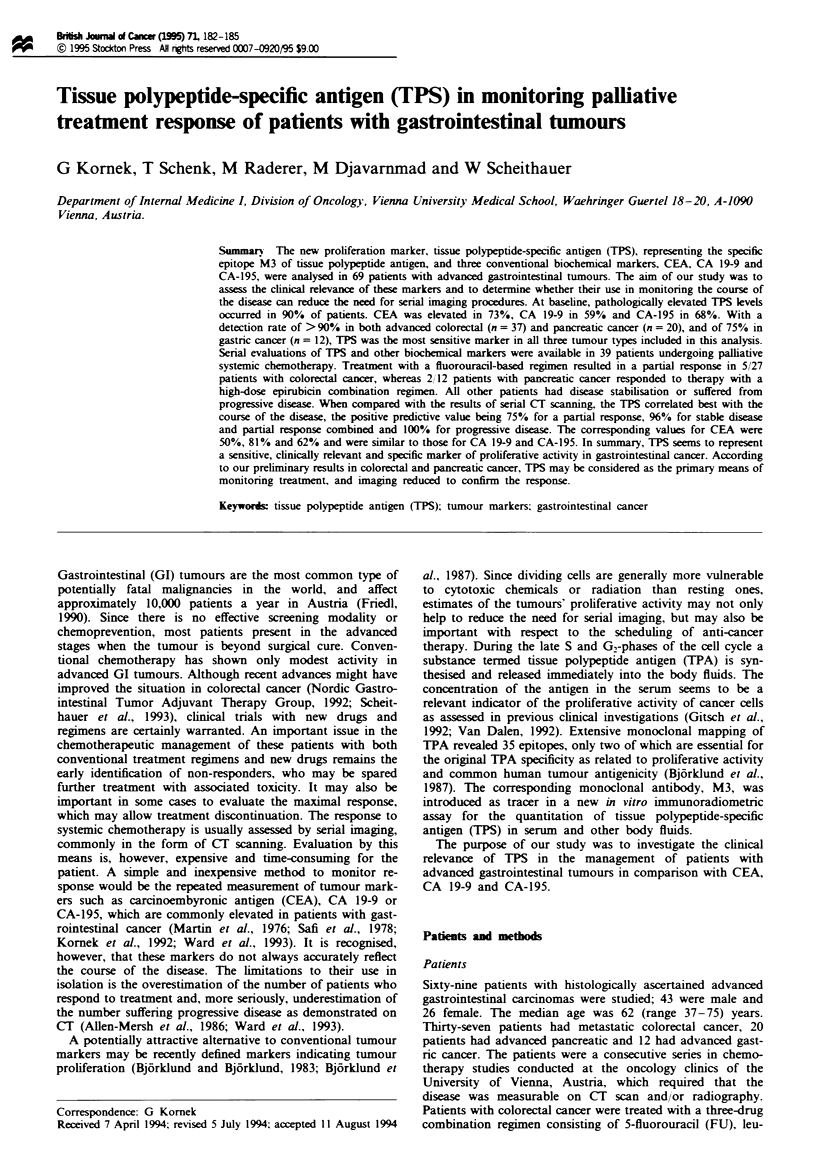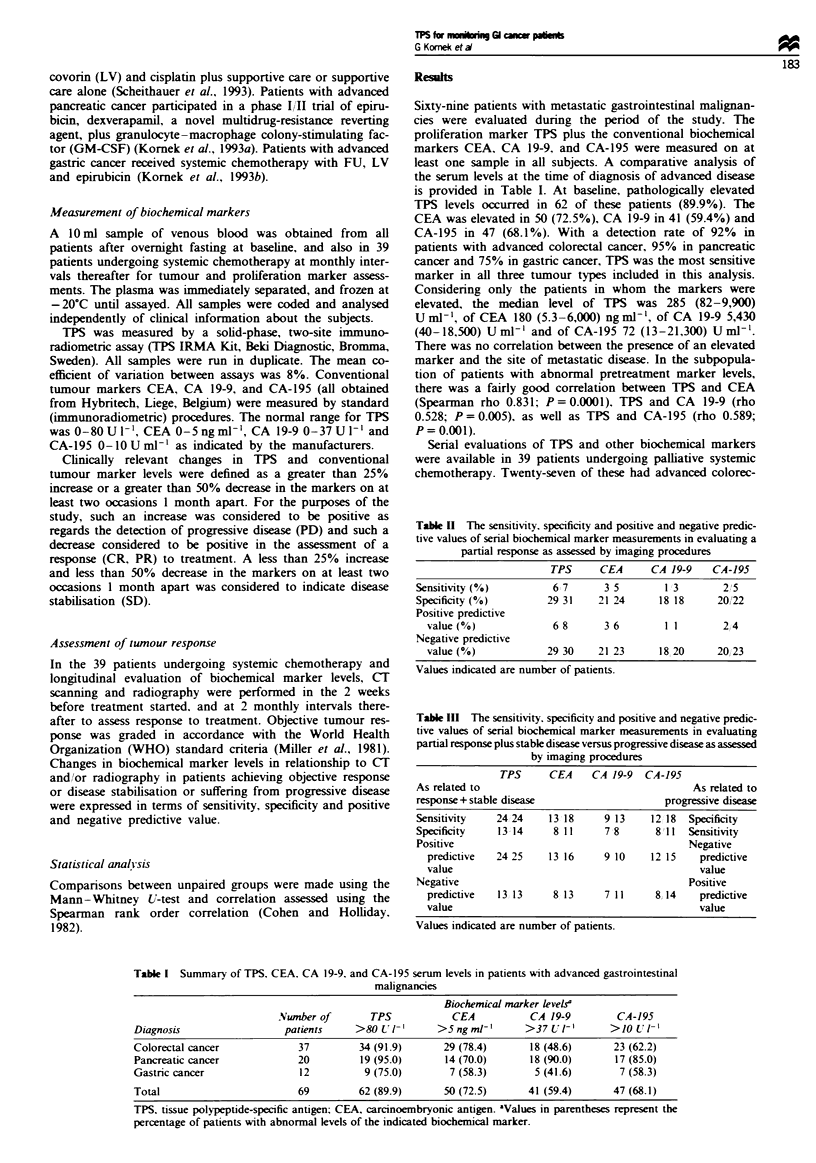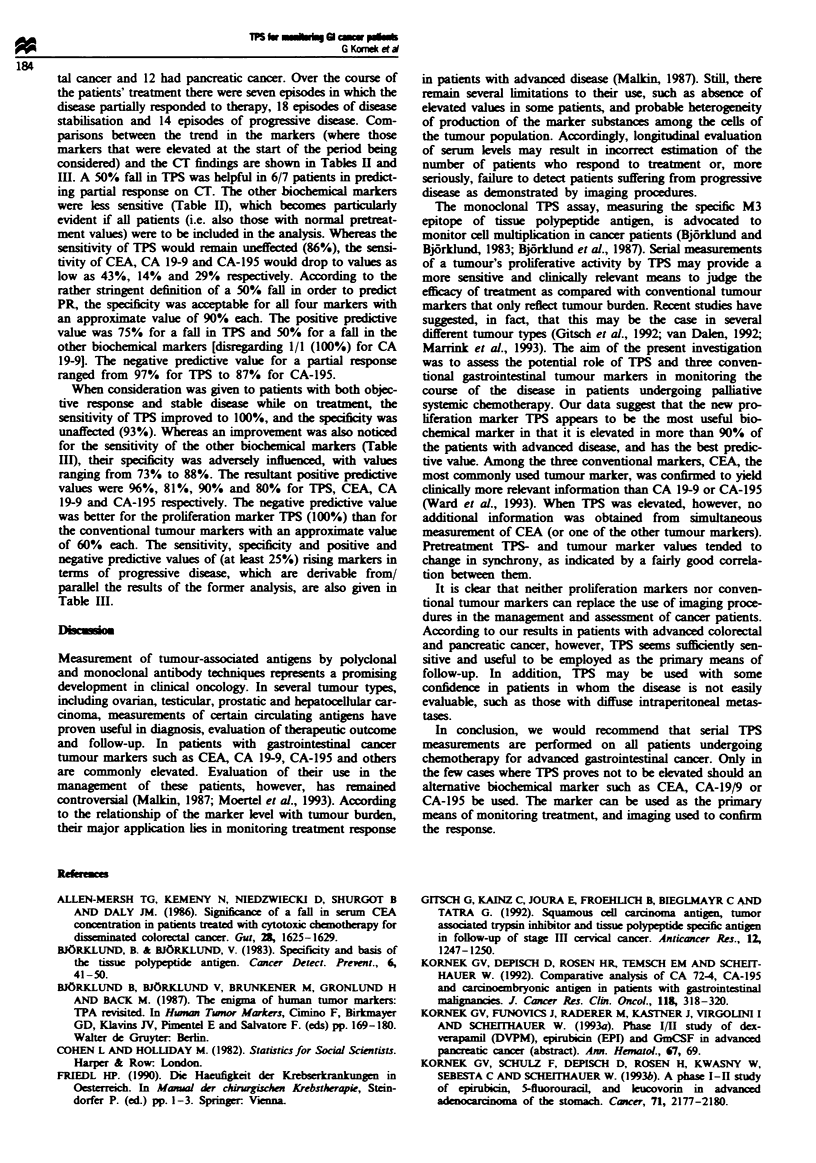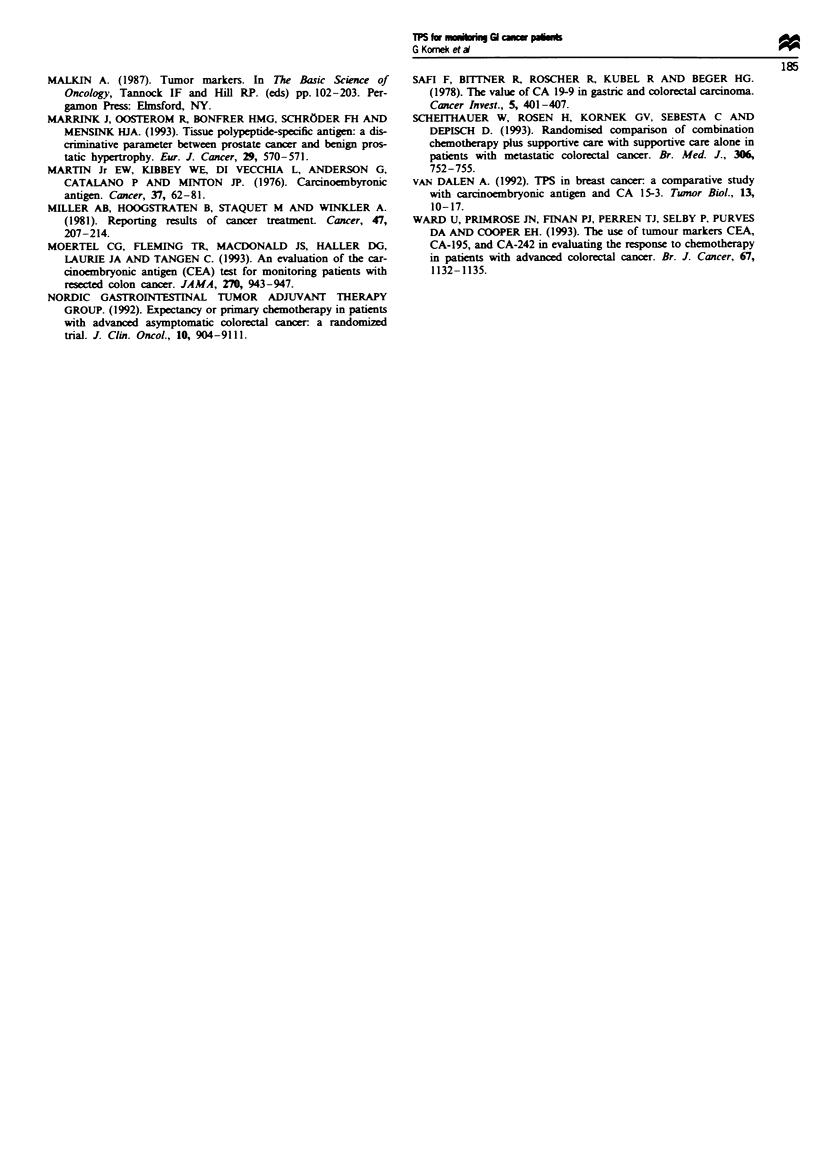# Tissue polypeptide-specific antigen (TPS) in monitoring palliative treatment response of patients with gastrointestinal tumours.

**DOI:** 10.1038/bjc.1995.37

**Published:** 1995-01

**Authors:** G. Kornek, T. Schenk, M. Raderer, M. Djavarnmad, W. Scheithauer

**Affiliations:** Department of Internal Medicine I, Vienna University Medical School, Austria.

## Abstract

The new proliferation marker, tissue polypeptide-specific antigen (TPS), representing the specific epitope M3 of tissue polypeptide antigen, and three conventional biochemical markers, CEA, CA 19-9 and CA-195, were analysed in 69 patients with advanced gastrointestinal tumours. The aim of our study was to assess the clinical relevance of these markers and to determine whether their use in monitoring the course of the disease can reduce the need for serial imaging procedures. At baseline, pathologically elevated TPS levels occurred in 90% of patients. CEA was elevated in 73%, CA 19-9 in 59% and CA-195 in 68%. With a detection rate of > 90% in both advanced colorectal (n = 37) and pancreatic cancer (n = 20), and of 75% in gastric cancer (n = 12), TPS was the most sensitive marker in all three tumour types included in this analysis. Serial evaluations of TPS and other biochemical markers were available in 39 patients undergoing palliative systemic chemotherapy. Treatment with a fluorouracil-based regimen resulted in a partial response in 5/27 patients with colorectal cancer, whereas 2/12 patients with pancreatic cancer responded to therapy with a high-dose epirubicin combination regimen. All other patients had disease stabilisation or suffered from progressive disease. When compared with the results of serial CT scanning, the TPS correlated best with the course of the disease, the positive predictive value being 75% for a partial response, 96% for stable disease and partial response combined and 100% for progressive disease. The corresponding values for CEA were 50%, 81% and 62% and were similar to those for CA 19-9 and CA-195. In summary, TPS seems to represent a sensitive, clinically relevant and specific marker of proliferative activity in gastrointestinal cancer. According to our preliminary results in colorectal and pancreatic cancer, TPS may be considered as the primary means of monitoring treatment, and imaging reduced to confirm the response.


British Journal of Cancer (1995) 71, 182–185

© 1995 Stockton Press All rights reserved 0007-0920/95 $9.00

### Tissue polypeptide-specific antigen (TPS) in monitoring palliative treatment response of patients with gastrointestinal tumours

G Kornek, T Schenk, M Raderer, M Djavarnmad and W Scheithauer

*Department of Internal Medicine I, Division of Oncology, Vienna University Medical School, Waehringer Guertel 18–20, A-1090 Vienna, Austria.*

**Summary** The new proliferation marker, tissue polypeptide-specific antigen (TPS), representing the specific epitope M3 of tissue polypeptide antigen, and three conventional biochemical markers, CEA, CA 19-9 and CA-195, were analysed in 69 patients with advanced gastrointestinal tumours. The aim of our study was to assess the clinical relevance of these markers and to determine whether their use in monitoring the course of the disease can reduce the need for serial imaging procedures. At baseline, pathologically elevated TPS levels occurred in 90% of patients. CEA was elevated in 73%, CA 19-9 in 59% and CA-195 in 68%. With a detection rate of >90% in both advanced colorectal ($n = 37$) and pancreatic cancer ($n = 20$), and of 75% in gastric cancer ($n = 12$), TPS was the most sensitive marker in all three tumour types included in this analysis. Serial evaluations of TPS and other biochemical markers were available in 39 patients undergoing palliative systemic chemotherapy. Treatment with a fluorouracil-based regimen resulted in a partial response in 5/27 patients with colorectal cancer, whereas 2/12 patients with pancreatic cancer responded to therapy with a high-dose epirubicin combination regimen. All other patients had disease stabilisation or suffered from progressive disease. When compared with the results of serial CT scanning, the TPS correlated best with the course of the disease, the positive predictive value being 75% for a partial response, 96% for stable disease and partial response combined and 100% for progressive disease. The corresponding values for CEA were 50%, 81% and 62% and were similar to those for CA 19-9 and CA-195. In summary, TPS seems to represent a sensitive, clinically relevant and specific marker of proliferative activity in gastrointestinal cancer. According to our preliminary results in colorectal and pancreatic cancer, TPS may be considered as the primary means of monitoring treatment, and imaging reduced to confirm the response.

**Keywords:** tissue polypeptide antigen (TPS); tumour markers; gastrointestinal cancer

Gastrointestinal (GI) tumours are the most common type of potentially fatal malignancies in the world, and affect approximately 10,000 patients a year in Austria (Friedl, 1990). Since there is no effective screening modality or chemoprevention, most patients present in the advanced stages when the tumour is beyond surgical cure. Conventional chemotherapy has shown only modest activity in advanced GI tumours. Although recent advances might have improved the situation in colorectal cancer (Nordic Gastrointestinal Tumor Adjuvant Therapy Group, 1992; Scheithauer *et al.*, 1993), clinical trials with new drugs and regimens are certainly warranted. An important issue in the chemotherapeutic management of these patients with both conventional treatment regimens and new drugs remains the early identification of non-responders, who may be spared further treatment with associated toxicity. It may also be important in some cases to evaluate the maximal response, which may allow treatment discontinuation. The response to systemic chemotherapy is usually assessed by serial imaging, commonly in the form of CT scanning. Evaluation by this means is, however, expensive and time-consuming for the patient. A simple and inexpensive method to monitor response would be the repeated measurement of tumour markers such as carcinoembyronic antigen (CEA), CA 19-9 or CA-195, which are commonly elevated in patients with gastrointestinal cancer (Martin *et al.*, 1976; Safi *et al.*, 1978; Kornek *et al.*, 1992; Ward *et al.*, 1993). It is recognised, however, that these markers do not always accurately reflect the course of the disease. The limitations to their use in isolation is the overestimation of the number of patients who respond to treatment and, more seriously, underestimation of the number suffering progressive disease as demonstrated on CT (Allen-Mersh *et al.*, 1986; Ward *et al.*, 1993).

A potentially attractive alternative to conventional tumour markers may be recently defined markers indicating tumour proliferation (Björklund and Björklund, 1983; Björklund *et al.*, 1987). Since dividing cells are generally more vulnerable to cytotoxic chemicals or radiation than resting ones, estimates of the tumours' proliferative activity may not only help to reduce the need for serial imaging, but may also be important with respect to the scheduling of anti-cancer therapy. During the late S and $G_2$-phases of the cell cycle a substance termed tissue polypeptide antigen (TPA) is synthesised and released immediately into the body fluids. The concentration of the antigen in the serum seems to be a relevant indicator of the proliferative activity of cancer cells as assessed in previous clinical investigations (Gitsch *et al.*, 1992; Van Dalen, 1992). Extensive monoclonal mapping of TPA revealed 35 epitopes, only two of which are essential for the original TPA specificity as related to proliferative activity and common human tumour antigenicity (Björklund *et al.*, 1987). The corresponding monoclonal antibody, M3, was introduced as tracer in a new *in vitro* immunoradiometric assay for the quantitation of tissue polypeptide-specific antigen (TPS) in serum and other body fluids.

The purpose of our study was to investigate the clinical relevance of TPS in the management of patients with advanced gastrointestinal tumours in comparison with CEA, CA 19-9 and CA-195.

### Patients and methods

### *Patients*

Sixty-nine patients with histologically ascertained advanced gastrointestinal carcinomas were studied; 43 were male and 26 female. The median age was 62 (range 37–75) years. Thirty-seven patients had metastatic colorectal cancer, 20 patients had advanced pancreatic and 12 had advanced gastric cancer. The patients were a consecutive series in chemotherapy studies conducted at the oncology clinics of the University of Vienna, Austria, which required that the disease was measurable on CT scan and/or radiography. Patients with colorectal cancer were treated with a three-drug combination regimen consisting of 5-fluorouracil (FU), leu-

Correspondence: G Kornek
Received 7 April 1994; revised 5 July 1994; accepted 11 August 1994

covorin (LV) and cisplatin plus supportive care or supportive care alone (Scheithauer *et al*., 1993). Patients with advanced pancreatic cancer participated in a phase I/II trial of epirubicin, dexverapamil, a novel multidrug-resistance reverting agent, plus granulocyte–macrophage colony-stimulating factor (GM-CSF) (Kornek *et al*., 1993*a*). Patients with advanced gastric cancer received systemic chemotherapy with FU, LV and epirubicin (Kornek *et al*., 1993*b*).

### *Measurement of biochemical markers*

A 10 ml sample of venous blood was obtained from all patients after overnight fasting at baseline, and also in 39 patients undergoing systemic chemotherapy at monthly intervals thereafter for tumour and proliferation marker assessments. The plasma was immediately separated, and frozen at −20°C until assayed. All samples were coded and analysed independently of clinical information about the subjects.

TPS was measured by a solid-phase, two-site immunoradiometric assay (TPS IRMA Kit, Beki Diagnostic, Bromma, Sweden). All samples were run in duplicate. The mean coefficient of variation between assays was 8%. Conventional tumour markers CEA, CA 19-9, and CA-195 (all obtained from Hybritech, Liege, Belgium) were measured by standard (immunoradiometric) procedures. The normal range for TPS was 0–80 U $l^{-1}$, CEA 0–5 ng $ml^{-1}$, CA 19-9 0–37 U $l^{-1}$ and CA-195 0–10 U $ml^{-1}$ as indicated by the manufacturers.

Clinically relevant changes in TPS and conventional tumour marker levels were defined as a greater than 25% increase or a greater than 50% decrease in the markers on at least two occasions 1 month apart. For the purposes of the study, such an increase was considered to be positive as regards the detection of progressive disease (PD) and such a decrease considered to be positive in the assessment of a response (CR, PR) to treatment. A less than 25% increase and less than 50% decrease in the markers on at least two occasions 1 month apart was considered to indicate disease stabilisation (SD).

### *Assessment of tumour response*

In the 39 patients undergoing systemic chemotherapy and longitudinal evaluation of biochemical marker levels, CT scanning and radiography were performed in the 2 weeks before treatment started, and at 2 monthly intervals thereafter to assess response to treatment. Objective tumour response was graded in accordance with the World Health Organization (WHO) standard criteria (Miller *et al*., 1981). Changes in biochemical marker levels in relationship to CT and/or radiography in patients achieving objective response or disease stabilisation or suffering from progressive disease were expressed in terms of sensitivity, specificity and positive and negative predictive value.

### *Statistical analysis*

Comparisons between unpaired groups were made using the Mann–Whitney *U*-test and correlation assessed using the Spearman rank order correlation (Cohen and Holliday, 1982).

### Results

Sixty-nine patients with metastatic gastrointestinal malignancies were evaluated during the period of the study. The proliferation marker TPS plus the conventional biochemical markers CEA, CA 19-9, and CA-195 were measured on at least one sample in all subjects. A comparative analysis of the serum levels at the time of diagnosis of advanced disease is provided in Table I. At baseline, pathologically elevated TPS levels occurred in 62 of these patients (89.9%). The CEA was elevated in 50 (72.5%), CA 19-9 in 41 (59.4%) and CA-195 in 47 (68.1%). With a detection rate of 92% in patients with advanced colorectal cancer, 95% in pancreatic cancer and 75% in gastric cancer, TPS was the most sensitive marker in all three tumour types included in this analysis. Considering only the patients in whom the markers were elevated, the median level of TPS was 285 (82–9,900) U $ml^{-1}$, of CEA 180 (5.3–6,000) ng $ml^{-1}$, of CA 19-9 5,430 (40–18,500) U $ml^{-1}$ and of CA-195 72 (13–21,300) U $ml^{-1}$. There was no correlation between the presence of an elevated marker and the site of metastatic disease. In the subpopulation of patients with abnormal pretreatment marker levels, there was a fairly good correlation between TPS and CEA (Spearman rho 0.831; $P = 0.0001$), TPS and CA 19-9 (rho 0.528; $P = 0.005$), as well as TPS and CA-195 (rho 0.589; $P = 0.001$).

Serial evaluations of TPS and other biochemical markers were available in 39 patients undergoing palliative systemic chemotherapy. Twenty-seven of these had advanced colorec-

**Table II** The sensitivity, specificity and positive and negative predictive values of serial biochemical marker measurements in evaluating a partial response as assessed by imaging procedures

| | *TPS* | *CEA* | *CA 19-9* | *CA-195* |
|---|---|---|---|---|
| Sensitivity (%) | 6/7 | 3/5 | 1/3 | 2/5 |
| Specificity (%) | 29/31 | 21/24 | 18/18 | 20/22 |
| Positive predictive value (%) | 6/8 | 3/6 | 1/1 | 2/4 |
| Negative predictive value (%) | 29/30 | 21/23 | 18/20 | 20/23 |

Values indicated are number of patients.

**Table III** The sensitivity, specificity and positive and negative predictive values of serial biochemical marker measurements in evaluating partial response plus stable disease versus progressive disease as assessed by imaging procedures

| As related to response + stable disease | *TPS* | *CEA* | *CA 19-9* | *CA-195* | As related to progressive disease |
|---|---|---|---|---|---|
| Sensitivity | 24/24 | 13/18 | 9/13 | 12/18 | Specificity |
| Specificity | 13/14 | 8/11 | 7/8 | 8/11 | Sensitivity |
| Positive predictive value | 24/25 | 13/16 | 9/10 | 12/15 | Negative predictive value |
| Negative predictive value | 13/13 | 8/13 | 7/11 | 8/14 | Positive predictive value |

Values indicated are number of patients.

**Table I** Summary of TPS, CEA, CA 19-9, and CA-195 serum levels in patients with advanced gastrointestinal malignancies

| | | Biochemical marker levels[a] | | | |
|---|---|---|---|---|---|
| *Diagnosis* | *Number of patients* | *TPS >80 U $l^{-1}$* | *CEA >5 ng $ml^{-1}$* | *CA 19-9 >37 U $l^{-1}$* | *CA-195 >10 U $l^{-1}$* |
| Colorectal cancer | 37 | 34 (91.9) | 29 (78.4) | 18 (48.6) | 23 (62.2) |
| Pancreatic cancer | 20 | 19 (95.0) | 14 (70.0) | 18 (90.0) | 17 (85.0) |
| Gastric cancer | 12 | 9 (75.0) | 7 (58.3) | 5 (41.6) | 7 (58.3) |
| Total | 69 | 62 (89.9) | 50 (72.5) | 41 (59.4) | 47 (68.1) |

TPS, tissue polypeptide-specific antigen; CEA, carcinoembryonic antigen. [a]Values in parentheses represent the percentage of patients with abnormal levels of the indicated biochemical marker.

tal cancer and 12 had pancreatic cancer. Over the course of the patients' treatment there were seven episodes in which the disease partially responded to therapy, 18 episodes of disease stabilisation and 14 episodes of progressive disease. Comparisons between the trend in the markers (where those markers that were elevated at the start of the period being considered) and the CT findings are shown in Tables II and III. A 50% fall in TPS was helpful in 6/7 patients in predicting partial response on CT. The other biochemical markers were less sensitive (Table II), which becomes particularly evident if all patients (i.e. also those with normal pretreatment values) were to be included in the analysis. Whereas the sensitivity of TPS would remain uneffected (86%), the sensitivity of CEA, CA 19-9 and CA-195 would drop to values as low as 43%, 14% and 29% respectively. According to the rather stringent definition of a 50% fall in order to predict PR, the specificity was acceptable for all four markers with an approximate value of 90% each. The positive predictive value was 75% for a fall in TPS and 50% for a fall in the other biochemical markers [disregarding 1/1 (100%) for CA 19-9]. The negative predictive value for a partial response ranged from 97% for TPS to 87% for CA-195.

When consideration was given to patients with both objective response and stable disease while on treatment, the sensitivity of TPS improved to 100%, and the specificity was unaffected (93%). Whereas an improvement was also noticed for the sensitivity of the other biochemical markers (Table III), their specificity was adversely influenced, with values ranging from 73% to 88%. The resultant positive predictive values were 96%, 81%, 90% and 80% for TPS, CEA, CA 19-9 and CA-195 respectively. The negative predictive value was better for the proliferation marker TPS (100%) than for the conventional tumour markers with an approximate value of 60% each. The sensitivity, specificity and positive and negative predictive values of (at least 25%) rising markers in terms of progressive disease, which are derivable from/parallel the results of the former analysis, are also given in Table III.

### Discussion

Measurement of tumour-associated antigens by polyclonal and monoclonal antibody techniques represents a promising development in clinical oncology. In several tumour types, including ovarian, testicular, prostatic and hepatocellular carcinoma, measurements of certain circulating antigens have proven useful in diagnosis, evaluation of therapeutic outcome and follow-up. In patients with gastrointestinal cancer tumour markers such as CEA, CA 19-9, CA-195 and others are commonly elevated. Evaluation of their use in the management of these patients, however, has remained controversial (Malkin, 1987; Moertel *et al.*, 1993). According to the relationship of the marker level with tumour burden, their major application lies in monitoring treatment response in patients with advanced disease (Malkin, 1987). Still, there remain several limitations to their use, such as absence of elevated values in some patients, and probable heterogeneity of production of the marker substances among the cells of the tumour population. Accordingly, longitudinal evaluation of serum levels may result in incorrect estimation of the number of patients who respond to treatment or, more seriously, failure to detect patients suffering from progressive disease as demonstrated by imaging procedures.

The monoclonal TPS assay, measuring the specific M3 epitope of tissue polypeptide antigen, is advocated to monitor cell multiplication in cancer patients (Björklund and Björklund, 1983; Björklund *et al.*, 1987). Serial measurements of a tumour's proliferative activity by TPS may provide a more sensitive and clinically relevant means to judge the efficacy of treatment as compared with conventional tumour markers that only reflect tumour burden. Recent studies have suggested, in fact, that this may be the case in several different tumour types (Gitsch *et al.*, 1992; van Dalen, 1992; Marrink *et al.*, 1993). The aim of the present investigation was to assess the potential role of TPS and three conventional gastrointestinal tumour markers in monitoring the course of the disease in patients undergoing palliative systemic chemotherapy. Our data suggest that the new proliferation marker TPS appears to be the most useful biochemical marker in that it is elevated in more than 90% of the patients with advanced disease, and has the best predictive value. Among the three conventional markers, CEA, the most commonly used tumour marker, was confirmed to yield clinically more relevant information than CA 19-9 or CA-195 (Ward *et al.*, 1993). When TPS was elevated, however, no additional information was obtained from simultaneous measurement of CEA (or one of the other tumour markers). Pretreatment TPS- and tumour marker values tended to change in synchrony, as indicated by a fairly good correlation between them.

It is clear that neither proliferation markers nor conventional tumour markers can replace the use of imaging procedures in the management and assessment of cancer patients. According to our results in patients with advanced colorectal and pancreatic cancer, however, TPS seems sufficiently sensitive and useful to be employed as the primary means of follow-up. In addition, TPS may be used with some confidence in patients in whom the disease is not easily evaluable, such as those with diffuse intraperitoneal metastases.

In conclusion, we would recommend that serial TPS measurements are performed on all patients undergoing chemotherapy for advanced gastrointestinal cancer. Only in the few cases where TPS proves not to be elevated should an alternative biochemical marker such as CEA, CA-19/9 or CA-195 be used. The marker can be used as the primary means of monitoring treatment, and imaging used to confirm the response.

### References

ALLEN-MERSH TG, KEMENY N, NIEDZWIECKI D, SHURGOT B AND DALY JM. (1986). Significance of a fall in serum CEA concentration in patients treated with cytotoxic chemotherapy for disseminated colorectal cancer. *Gut*, **28**, 1625–1629.

BJÖRKLUND, B. & BJÖRKLUND, V. (1983). Specificity and basis of the tissue polypeptide antigen. *Cancer Detect. Prevent.*, **6**, 41–50.

BJÖRKLUND B, BJÖRKLUND V, BRUNKENER M, GRONLUND H AND BACK M. (1987). The enigma of human tumor markers: TPA revisited. In *Human Tumor Markers*, Cimino F, Birkmayer GD, Klavins JV, Pimentel E and Salvatore F. (eds) pp. 169–180. Walter de Gruyter: Berlin.

COHEN L AND HOLLIDAY M. (1982). *Statistics for Social Scientists*. Harper & Row: London.

FRIEDL HP. (1990). Die Haeufigkeit der Krebserkrankungen in Oesterreich. In *Manual der chirurgischen Krebstherapie*, Steindorfer P. (ed.) pp. 1–3. Springer: Vienna.

GITSCH G, KAINZ C, JOURA E, FROEHLICH B, BIEGLMAYR C AND TATRA G. (1992). Squamous cell carcinoma antigen, tumor associated trypsin inhibitor and tissue polypeptide specific antigen in follow-up of stage III cervical cancer. *Anticancer Res.*, **12**, 1247–1250.

KORNEK GV, DEPISCH D, ROSEN HR, TEMSCH EM AND SCHEITHAUER W. (1992). Comparative analysis of CA 72-4, CA-195 and carcinoembryonic antigen in patients with gastrointestinal malignancies. *J. Cancer Res. Clin. Oncol.*, **118**, 318–320.

KORNEK GV, FUNOVICS J, RADERER M, KASTNER J, VIRGOLINI I AND SCHEITHAUER W. (1993*a*). Phase I/II study of dexverapamil (DVPM), epirubicin (EPI) and GmCSF in advanced pancreatic cancer (abstract). *Ann. Hematol.*, **67**, 69.

KORNEK GV, SCHULZ F, DEPISCH D, ROSEN H, KWASNY W, SEBESTA C AND SCHEITHAUER W. (1993*b*). A phase I–II study of epirubicin, 5-fluorouracil, and leucovorin in advanced adenocarcinoma of the stomach. *Cancer*, **71**, 2177–2180.

MALKIN A. (1987). Tumor markers. In *The Basic Science of Oncology*, Tannock IF and Hill RP. (eds) pp. 102–203. Pergamon Press: Elmsford, NY.

MARRINK J, OOSTEROM R, BONFRER HMG, SCHRÖDER FH AND MENSINK HJA. (1993). Tissue polypeptide-specific antigen: a discriminative parameter between prostate cancer and benign prostatic hypertrophy. *Eur. J. Cancer*, **29**, 570–571.

MARTIN Jr EW, KIBBEY WE, DI VECCHIA L, ANDERSON G, CATALANO P AND MINTON JP. (1976). Carcinoembyronic antigen. *Cancer*, **37**, 62–81.

MILLER AB, HOOGSTRATEN B, STAQUET M AND WINKLER A. (1981). Reporting results of cancer treatment. *Cancer*, **47**, 207–214.

MOERTEL CG, FLEMING TR, MACDONALD JS, HALLER DG, LAURIE JA AND TANGEN C. (1993). An evaluation of the carcinoembryonic antigen (CEA) test for monitoring patients with resected colon cancer. *JAMA*, **270**, 943–947.

NORDIC GASTROINTESTINAL TUMOR ADJUVANT THERAPY GROUP. (1992). Expectancy or primary chemotherapy in patients with advanced asymptomatic colorectal cancer: a randomized trial. *J. Clin. Oncol.*, **10**, 904–9111.

SAFI F, BITTNER R, ROSCHER R, KUBEL R AND BEGER HG. (1978). The value of CA 19-9 in gastric and colorectal carcinoma. *Cancer Invest.*, **5**, 401–407.

SCHEITHAUER W, ROSEN H, KORNEK GV, SEBESTA C AND DEPISCH D. (1993). Randomised comparison of combination chemotherapy plus supportive care with supportive care alone in patients with metastatic colorectal cancer. *Br. Med. J.*, **306**, 752–755.

VAN DALEN A. (1992). TPS in breast cancer: a comparative study with carcinoembryonic antigen and CA 15-3. *Tumor Biol.*, **13**, 10–17.

WARD U, PRIMROSE JN, FINAN PJ, PERREN TJ, SELBY P, PURVES DA AND COOPER EH. (1993). The use of tumour markers CEA, CA-195, and CA-242 in evaluating the response to chemotherapy in patients with advanced colorectal cancer. *Br. J. Cancer*, **67**, 1132–1135.